# GDF15 is a putative biomarker for distinguishing pulmonary veno-occlusive disease and pulmonary arterial hypertension

**DOI:** 10.1172/JCI199159

**Published:** 2025-11-20

**Authors:** Amit Prabhakar, Eckart M.D.D. De Bie, Jacqueline T. DesJardin, Prajakta Ghatpande, Stefan Gräf, Luke S. Howard, S. John Wort, Colin Church, David G. Kiely, UK Pulmonary Arterial Hypertension Cohort Study Consortium, Emily Sumpena, Thin Aung, Shenrae Carter, Jasleen Kukreja, Steven Hays, John R. Greenland, Jonathan P. Singer, Michael Wax, Paul J. Wolters, Marc A. Simon, Mark Toshner, Giorgio Lagna, Akiko Hata

**Affiliations:** 1 Cardiovascular Research Institute, UCSF, San Francisco, California, USA.; 2 Victor Phillip Dahdaleh Heart & Lung Research Institute, University of Cambridge, Cambridge, United Kingdom.; 3 Royal Papworth Hospital NHS Foundation Trust, Cambridge, United Kingdom.; 4 Pulmonary Vascular Disease, PAH Center of Comprehensive Care, Division of Cardiology, Department of Medicine, UCSF, San Francisco, California, USA.; 5 Imperial College London Faculty of Medicine, National Heart & Lung Institute, London, United Kingdom.; 6 Hammersmith Hospital, Imperial College Healthcare NHS Trust, London, United Kingdom.; 7 Royal Brompton Hospital, Adult Centre for Pulmonary Hypertension, London, United Kingdom.; 8 Golden Jubilee National University Hospital and University of Glasgow, Glasgow, United Kingdom.; 9 Sheffield Pulmonary Vascular Disease Unit and NIHR Biomedical Research Centre, Sheffield Teaching Hospitals NHS Foundation Trust, UK and Clinical Medicine, University of Sheffield, Sheffield, United Kingdom.; 10 United Kingdom National Cohort Study of Idiopathic and Heritable Pulmonary Arterial Hypertension Consortium is detailed in supplemental materials.; 11 Pulmonary, Critical Care, Allergy and Sleep Medicine, UCSF, San Francisco, California, USA.; 12 Department of Cardiothoracic Surgery, UCSF, San Francisco, California, USA.; 13 San Francisco VA Health Care System, San Francisco, California, USA.; 14 Department of Biochemistry and Biophysics, UCSF, San Francisco, California, USA.

**Keywords:** Clinical Research, Vascular biology, Biomarkers, Cardiovascular disease, Hypertension

## Abstract

Study identifies GDF15 as a biomarker—accurately distinguishing PVOD from other PAH forms and predicting outcomes across PVOD, IPAH, and HPAH—advancing earlier diagnosis and personalized treatment.

**To the Editor:** Pulmonary veno-occlusive disease (PVOD) is a rare, severe group 1 pulmonary arterial hypertension (PAH) subtype with poor survival (1). PAH-targeted vasodilators can cause life-threatening pulmonary edema in PVOD, underscoring the need for diagnostic tools to distinguish it from other PAH subtypes (1). In patients and mitomycin C–induced (MMC-induced) model animals, aberrant activation of integrated stress response (ISR) via protein kinase R (PKR) drives cardiovascular phenotypes of PVOD (2–4). Inhibition of the PKR-ISR axis, using either the PKR inhibitor C16 or the ISR inhibitor ISRIB, reverses PVOD phenotypes (2–4). Because GDF15 is a cytokine induced by the ISR (5), we compared plasma GDF15 levels in rat models of PVOD and PAH to evaluate its potential as a biomarker. Upon MMC treatment, GDF15 mRNA and plasma protein levels increased (Supplemental Figure 1A; supplemental material available online with this article; https://doi.org/10.1172/JCI199159DS1), and ISRIB reversed treatment reserved this effect (6) (Supplemental Figure 1B). Plasma GDF15 levels were 2.2-fold higher in PVOD model rats compared with those in monocrotaline-induced PAH model rats (Supplemental Figure 1C), suggesting a potential distinction in circulating GDF15 levels between PVOD and other PAH subtypes.

Plasma GDF15 concentrations in patients with PVOD, idiopathic PAH (IPAH), heritable PAH (HPAH), group 2–4 PH (Other PH), and chronic obstructive pulmonary disease without PH (COPD) as well as individuals acting as healthy controls were quantified (Figure 1A). The median GDF15 level in the PVOD group was 12.4-, 3.0-, 2.4-, 2.1-, and 2.4-fold higher than that in the control, IPAH, HPAH, Other PH, and COPD groups, respectively (Figure 1A). No significant sex-dependent differences in GDF15 were observed (Supplemental Figure 2A). ROC analysis showed that the PVOD group had the highest AUC among all cohorts, with both sensitivity and specificity of 100% (Figure 1B), whereas specificity for IPAH, HPAH, Other PH, and COPD groups was lower at the same sensitivity (Figure 1C). It also showed that plasma GDF15 levels distinguished the PVOD group from other cohorts, with AUC values of 94% or higher (Figure 1D). At 100% specificity for PVOD, GDF15 maintained a specificity greater than 58% when compared with other cohorts (Figure 1, D–F). GDF15 also distinguished the PVOD cohort from the combined IPAH, HPAH, and Other PH cohort and the combined IPAH and HPAH cohort, with an optimal cutoff value of 1,658 pg/mL and 81% sensitivity and 98% specificity. GDF15 levels were significantly associated with PVOD compared with all reference cohorts (Figure 1E). An inverse correlation was observed between age-adjusted GDF15 and 6-minute walk distance (6MWD) in the combined PVOD, IPAH, and HPAH cohort, indicating an association with disease severity (Figure 1G); however, no such correlation was observed in the PVOD-alone cohort (Supplemental Figure 2B). Age-adjusted GDF15 (Figure 1H), but not other parameters (Figure 1, I–M), differentiated PVOD from IPAH and HPAH. Higher levels of GDF15 were associated with poorer survival in the combined PVOD, IPAH, and HPAH cohort (Figure 1N), with a similar trend observed in the PVOD cohort (Supplemental Figure 2C). Our findings suggest that GDF15 may serve as both a diagnostic biomarker to distinguish PVOD from other PAH subtypes and a prognostic biomarker for patients with PVOD, IPAH, and HPAH.

For detailed methods, information regarding study population characteristics (Supplemental Table 1), methods, sex as a biological variable, statistics, study approval, author contributions, and acknowledgments, see the supplemental materials.

## Funding support

This work is the result of NIH funding, in whole or in part, and is subject to the NIH Public Access Policy. Through acceptance of this federal funding, the NIH has been given a right to make the work publicly available in PubMed Central.

- NIH (T32HL007731 to JTD; R01AG058659 to MAS; K24HL174231 to JPS; HL151552, VA CX002011, and CFF HAYS19AB3 to JRG; and R01HL164581 and R01HL153915 to AH).
- Gates Cambridge Trust (OPP1144 to EMDDDB).
- National Institute for Health and Care Research (NIHR) (NIHR203321 to DGK).
- United Kingdom National Cohort Study of Idiopathic and Heritable Pulmonary Arterial Hypertension Consortium grant to LSH.
- Cardiorespiratory NIHR Cambridge Bioresource Centre to MT.
- British Heart Foundation (SP/12/12/29836 and SP/18/10/33975 to the UK Pulmonary Arterial Hypertension Cohort Study Consortium).
- Nina Ireland Program for Lung Health to PJW.

## Supplementary Material

Supplemental data

Supporting data values

## Figures and Tables

**Figure 1:**
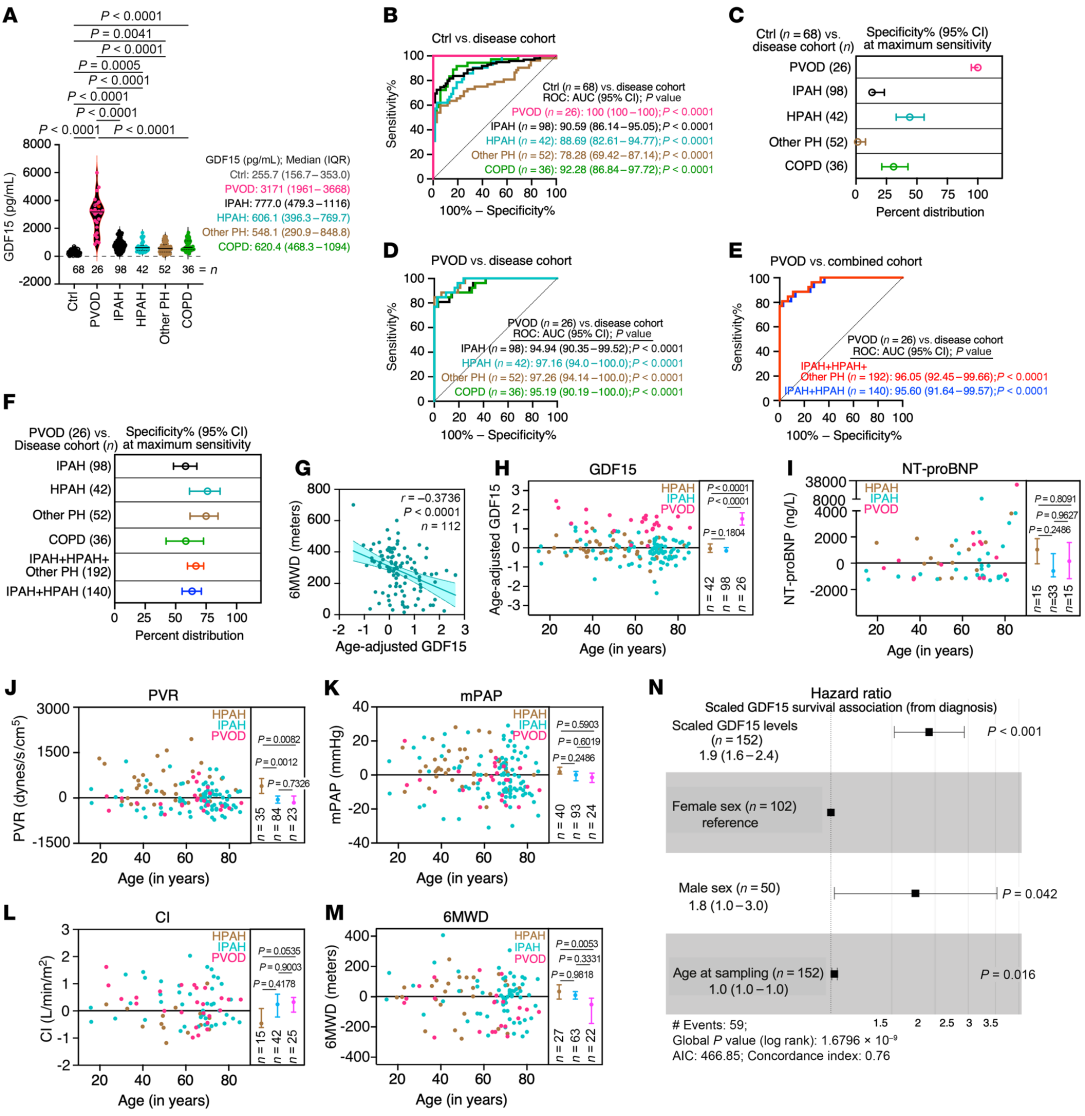
Plasma GDF15 as a diagnostic and prognostic biomarker for PVOD. (**A**) Violin plot of plasma GDF15 concentrations across different cohorts. *P* < 0.0001 by 1-way ANOVA with Tukey’s post hoc test. (**B**) ROC curves comparing GDF15 levels between a cohort of individuals acting as healthy controls (Ctrl) and other specified cohorts. (**C**) Specificity% (95% CI) at 100% sensitivity for distinguishing the control cohort from other specified cohorts. (**D**) ROC curves for distinguishing PVOD from individual cohorts. (**E**) ROC curves comparing PVOD with combined cohorts: IPAH, HPAH, and Other PH as well as IPAH and HPAH. (**F**) Specificity% (95% CI) at 100% sensitivity for distinguishing PVOD from individual or combined cohorts. Numbers associated with individual cohorts indicate the number of individuals within that cohort. (**G**) Pearson correlation between age-adjusted GDF15 and 6MWD in a combined cohort: PVOD, IPAH, and HPAH. (**H**–**M**) Standardized distributions of diagnostic parameters: age-adjusted GDF15 (**H**), N-terminal pro-B-type natriuretic peptide (NT-proBNP) (**I**), pulmonary vascular resistance (PVR) (**J**), mean pulmonary arterial pressure (mPAP) (**K**), cardiac index (CI) (**L**), and 6MWD (**M**) in PVOD, IPAH, and HPAH. Left: Individual values normalized to the median (*y* = 0). Right: Median values with 95% CI. (**N**) Cox proportional hazards model showing the association between plasma GDF15 and transplant-free survival in the combined cohort, PVOD, IPAH, and HPAH, adjusted for age and sex. Statistical analysis was performed using *C* statistics to calculate the AUC, followed by *Z* tests for pairwise comparisons (**B**, **D**, and **E**). Mixed-effects models with Tukey’s multiple comparisons test were used for analyses shown in panels **H** and **I**. *P* value < 0.05 was considered significant.

## Version 1 11/20/2025

In-Press Preview

## Version 2 01/16/2026

Electronic publication
