# Supplementary material for: GDF15 is a putative biomarker for distinguishing pulmonary veno-occlusive disease and pulmonary arterial hypertension
Source: J Clin Invest. 2025 Nov 20;136(2):e199159. doi: 10.1172/JCI199159 (PMC12807463; doi:10.1172/JCI199159)
Supplement: Supplemental data [file jci-136-199159-s067.pdf]

GDF15 as a Biomarker for Distinguishing Pulmonary Veno-Occlusive Disease and Pulmonary Arterial Hypertension

Amit Prabhakar[1], Eckart MDD De Bie[2,3], Jacqueline DesJardin[4], Prajakta Ghatpande[1], Stefan Gräf[2], Luke S. Howard[5,6], S. John Wort[5,7], Colin Church[8], David G. Kiely[9], UK Pulmonary Arterial Hypertension Cohort Study Consortium, Emily Sumpena[4], Thin Aung[4], Shenrae Carter[10], Jasleen Kukreja[10], Steven Hays[10], John R. Greenland[10,11], Jonathan P. Singer[10], Michael Wax[10], Paul J. Wolters[10], Marc A. Simon[4], Mark Toshner[2,3], Giorgio Lagna[1], Akiko Hata[1,12]

Address correspondence to: Akiko Hata, 555 Mission Bay Blvd. South, SCVRB Rm 252T, San Francisco, CA 94143, USA. Phone: 415-476-9758;
Email: akiko.hata@ucsf.edu

Supplementary Data:

- Characteristics of the study population
- Methods
- Author Contributions
- Acknowledgement
- Reference
- Supplementary Table 1 Demographic and clinical characteristics of study participants
- Supplementary Figure 1 Elevated plasma levels of GDF15 in an animal model of PVOD
- Supplementary Figure 2 Evaluating the potential of GDF15 as a diagnostic and prognostic biomarker for PVOD

Characteristics of the study population

The demographic and clinical characteristics of the study subjects, whose plasma samples were analyzed, are presented in Supplementary Table 1. The clinical presentation of the participants was heterogeneous and predominantly categorized into the following groups: Ctrl (n = 68), PVOD (n = 26), IPAH (n = 98), HPAH (n = 42), and all other types of PAH and PH, which includes group 1 PAH excluding PVOD, IPAH, and HPAH, as well as group 2-4 pulmonary hypertension (PH) (hereafter referred to as Other PH; n = 52), and COPD/emphysema with no clinical evidence of PH (hereafter referred to as COPD; *n* = 36). COPD patients are included in our study because elevated levels of plasma GDF15 have been reported in COPD (1-7). The pathological status of patients suspected of having PVOD, IPAH, HPAH, Other PH, or COPD was confirmed with a specialist center diagnosis before their inclusion in the study.

Methods

Reagents, kits, antibodies, PCR primers, instruments, and software used in the study are listed here:

(i) Reagents

no.	Reagent	Company	Catalog no.
01.	C16	Sigma-Aldrich	I9785
02.	DNase I	Ambion	AM2238
03.	Dynabeads protein A	Invitrogen	10002D
04.	Dynabeads protein G	Invitrogen	10004D
05.	rhGDF15	R&D systems	8146-GD/CF
06.	Mouse/Rat GDF15 ELISA	R&D systems	MGD150
07.	Human GDF15 ELISA (DuoSet)	R&D systems	DY957
08.	ISRIB	Sigma-Aldrich	SML0843
09.	Mitomycin C	Sigma-Aldrich	M0503
10.	Proteinase K	Invitrogen	P/N100005393

11.	Protease Inhibitor	Sigma	P8340
12.	Phosphatase Inhibitor	Sigma	P5726
13.	RNase inhibitors	Invitrogen	AM2696
14.	Monocrotaline	Sigma-Aldrich	C2401

(ii) Antibodies

no.	Antigen	Dilutions	Company	Catalog no.
01.	ATF4	1:250	Cell signaling Technology	11815
02.	CHOP	1:250	Cell signaling Technology	2895

(iii) RT-PCR primer sequences

Primer Name	Primer Sequence	Annotation
rGDF15-qPCR-F	5'-GCTGAGGTTCCTGCTGTT-3'	qRT-PCR for rat GDF15
rGDF15-qPCR-R	5'-GTTGAGTTGGGACTCAGAGAG-3'	
rGapdh-qPCR-F	5'-TGGATAGGGTGGCCGAAGTA-3'	qRT-PCR for rat GAPDH
rGapdh-qPCR-R	5'-GGAAACCCTGCCATCCATCA-3'	

(iv) Instruments and software

no.	Instrument/ software	Experiment	Company	Model no./ version no.
01.	Bioruptor	Sonication	Diagenode	Bioruptor Pico
02.	Dismembrator/Sonicator	Sonication	Fisher Scientific	550 sonic dismembrator
03.	Tissue lyser	Tissue lysis	Qiagen	TissueLyser II
04.	RT-PCR machine	qRT-PCR	BioRad	CFX connect
05.	SpectraMax	Absorbance, Fluorescence	Molecular Devices LLC	SpectraMax M
06.	NanoDrop spectrometer	Protein, DNA, and RNA quantitation	Thermo Scientific	NanoDrop 2000c
07.	MS Excel	Statistical analysis	Microsoft	MS office 365
08.	GraphPad Prism	Statistical analysis	GraphPad	Prism 10.3.1
09.	SPSS	Statistical analysis	IBM	SPSS 21.0
10.	R	Statistical analysis	The R foundation	Version 4.3.2
11.	Genomics Viewer	RNA-seq	Broad Institute	Integrative Genomics Viewer 2.4.14

Study Participants and Sample Correction

Plasma samples and clinical data from the UK National Cohort Study of Idiopathic and Heritable Pulmonary Arterial Hypertension (NCS), the UCSF PH Biobank (PHB), and the UCSF Lung Transplant Registry (LTR) were used in the study. The NCS provided samples of patients with IPAH (*n*= 88), HPAH (*n*= 42), PVOD (*n*= 19), Other G1 PAH (*n*= 1), and G2 PH (*n*= 1) aged 18–85 years who were recruited between February 19, 2014, and December 2021 from eight centers participating in the NCS (Clinicaltrials.gov: NCT01907295). Unrelated volunteers aged 18–72 years (n = 55) who self-declared as healthy and without a history of cardiovascular or respiratory disease were recruited to the NCS over the same period from the same centers. The PHB provided samples of patients with IPAH (*n* = 7), HPAH (*n* = 1), PVOD (*n* = 4), Other G1 PAH (*n* = 28), and G2-4 PH (*n* = 16) aged 23–84 years recruited from February 2023 till the present. All patients with PVOD are treated with either vasodilators or calcium channel blockers. Unrelated volunteers aged 21–68 years (*n* =15) who self-declared as healthy and without a history of cardiovascular or respiratory disease were recruited to the PHB over the same period from the same centers. The LTR provided samples of patients with IPAH (*n* = 3), PVOD (*n* = 3), other Group 1 PAH (*n* = 6), and COPD (*n* = 36), aged 32–74 years, who were recruited to the LTR from March 2013 to the present.

The diagnostic criteria for IPAH, HPAH, and PVOD used in this study followed contemporary international guidelines. Resting mean pulmonary arterial pressure and

pulmonary arterial wedge pressure were measured at routine clinical appointment visits. The diagnosis was made by a team of multidisciplinary specialists, including radiologists, cardiologists, and cardiothoracic surgeons. Clinical, functional, and hemodynamic characteristics at the time of PAH or PVOD diagnosis were both prospectively and retrospectively entered the database. The date of diagnosis corresponded to that of confirmatory right heart catheterization.

The diagnostic criteria for COPD patients followed the Global Initiative for Chronic Obstructive Lung Disease diagnostic criteria (8). Patients with COPD and clinical evidence of PH were excluded. All cases were diagnosed between March 1994 and December 2021, and diagnostic classification was made according to international guidelines(9). The whole-blood sample collection was performed with the participants' written informed consent for use in gene identification studies (UK Research Ethics Committee: 08/H0802/32) and processed as described previously (10). Blood samples for research were collected in tubes containing EDTA. Plasma samples were subjected to centrifugation and stored at –80 °C before use.

Study Approval

The animal protocol (AN200674-00D) was approved by the Institutional Animal Care and Use Committee (IACUC) at UCSF, and all experiments were conducted in accordance with the IACUC guidelines. The human study protocol UK REC Ref. 13/EE/0203 for the NCS was approved by the East of England Research Ethics

Committee, and protocols #22-36931 and #13-10738 for the PHB and LTR, respectively, were approved by the Institutional Review Board (IRB) at UCSF. The protocol for the gene identification studies #08/H0802/32 for the NCS was approved by the UK Research Ethics Committee.

Enzyme-Linked Immunosorbent Assay (ELISA) for GDF15

The amount of GDF15 protein in the rat plasma and patients' plasma samples was quantitated using the GDF15 Quantikine ELISA kit (R&D systems, DGD150) and GDF15 DuoSet ELISA kit (R&D systems, DY957) according to the manufacturer's protocol. The Bradford protein estimation method quantitated the relative concentrations of total proteins in the plasma, and the total protein amount adjusted the sample volume. The plasma samples were diluted at 1:10 in PBS before applying to pre-coated ELISA plates. Plates were washed with PBS-T three times and incubated with respective secondary antibodies conjugated to streptavidin-horseradish peroxide at room temperature (R.T.) for two hours. The ELISA was developed in a dark room at R.T. with a colorimetric substrate comprising tetramethylbenzidine and stabilized hydrogen peroxide mixed in equal volumes. The absorbance was measured at 450 nm. Plasma GDF15 concentrations were reported in pg/mL, and *P*-values were evaluated.

Sex as a biological variable

Sex was not considered a biological variable in this study. Both male and female animals were used in all experiments.

Animal care and use

Sprague-Dawley 8-9 weeks-old rats were housed at the ambient temperature of 25±2 °C and 50±5% relative humidity under 12/12 hours of dark/light cycle in the institutional animal facility.

PVOD rat model

Animals were housed in the vivarium of the cardiovascular research building at UCSF (San Francisco, USA). Both male and female Sprague Dawley rats (8-9 weeks old) were subjected to the following protocols to examine the effect of MMC and/or small molecule ISR inhibitor, ISRIB, or PKR inhibitor, C16. MMC was made by dissolving 2 mg MMC in 1 mL saline and was delivered to rats at 3 mg/kg dosage through intraperitoneal injection (i.p.) injections. Saline was used as a vehicle solution for MMC treatment. ISRIB solution was made by dissolving 5 mg ISRIB in 1 mL of dimethyl sulfoxide (DMSO), followed by dilution to 1 mg/mL, and was delivered to rats at 0.25 mg/kg dosage through i.p. injections. The vehicle solution consisted of 1 mL DMSO and 4 mL saline. C16 solution was made by dissolving 10 mg C16 in 1 mL DMSO, followed by dilution to a final concentration of 100 μg/mL, and was delivered to rats at 33.5 μg/kg dosage through i.p. injections. The vehicle solution consisted of 100 μL DMSO and 10 mL saline.

Protocol #1 MMC treatment: Rats were randomly divided into MMC (3 mg/kg) or saline (vehicle)--exposed groups. MMC or saline was administered once intraperitoneal injection (i.p.) on day 0 (d0). Rats were euthanized 24 days after the MMC/vehicle injection (d24). Hemodynamic measurements were made, RV hypertrophy was assessed, and tissue samples were collected.

Protocol #2 Delayed ISRIB treatment on d8 for 8 or 16 days: Rats were given MMC (3 mg/kg) or vehicle (saline) by i.p. on d0. For 16-day treatment, ISRIB (0.25 mg/kg) or vehicle (DMSO) was given three times a week between d8 and d24 (total of six injections) and euthanized on d24. For 8-day treatment, rats were given ISRIB (0.25 mg/kg) or vehicle three times a week between d8 and d16 (total of three injections) and euthanized on d24. Hemodynamic measurements were made, right ventricular hypertrophy was assessed, and tissue samples were collected.

Protocol #3 Delayed ISRIB treatment on d24 for 8 days: Rats were given MMC (3 mg/kg) or vehicle (saline) by i.p. on d0. Rats were given ISRIB (0.25 mg/kg) or vehicle three injections between d24 and d32 and euthanized on d32. Hemodynamic measurements were made, right ventricular hypertrophy was assessed, and tissue samples were collected.

Monocrotaline (MCT)-induced PAH rat model

MCT was dissolved in 0.5 N HCl, adjusted to pH 7.4 with 0.5 N NaOH, and diluted with sterile water to a concentration of 60 mg/mL. MCT (60 mg/kg) or vehicle (PBS) was subcutaneously injected into the ventral thorax of rats. The animals were maintained on a 12/12-hours light/dark cycle at 18–20 °C and 40–50% humidity for 32 days (food and water were provided ad libitum, and the animals were checked once daily), followed by assessment of the phenotypes.

Quantitative RT-PCR (qRT-PCR)

Total RNA was isolated from the rat lung tissue and subjected to cDNA preparation by the reverse transcription reaction with a commercial kit. qPCR analysis was performed in triplicate with a commercial kit. The relative expression values were determined by normalization to *GAPDH* transcript levels and calculated using the ΔΔCT method. qRT-PCR primer sequences and the kits are found in Supplementary Information.

Scripts for the analysis

The scripts used to analyze the association between GDF15 and patient outcomes are archived on GitHub (https://github.com/EckartDeBie/PVOD_analyses).

Statistical Analysis

Statistical analyses and graphical presentations were performed using GraphPad PRISM 10, and IBM SPSS 21.0. The primary analysis aimed to assess the interaction between plasma GDF15 levels and PVOD events. This was conducted by comparing the median plasma concentrations of GDF15 between individuals who developed PVOD, healthy controls, and individuals who developed PH using the Kruskal-Wallis test followed by Dunn's multiple comparison test. The results are presented as the absolute difference in median values. Data are reported as median with interquartile range (IQR), mean with standard error (SEM), or mean with 95% CI, as appropriate. To evaluate the diagnostic performance of plasma GDF15 in association with PVOD events, we conducted an ROC analysis. Sensitivity (true positive rate) was plotted against 100-specificity (true negative rate), and the AUC was used as an index of association, where a higher AUC indicates a stronger relationship. Sensitivity and specificity were determined at the optimal probability. The GDF15 optimal diagnostic cutoff value was determined using Youden's J statistic from the ROC curve and respective sensitivity and specificity values. To examine potential relationships between plasma GDF15 levels and other factors, Pearson's correlation analysis was performed to assess the dependence of age-adjusted GDF15 levels on specific manifestation types. Distribution analysis was performed using a mixed-effect model to compare studied parameters among PVOD, IPAH, and HPAH, where individual observations were displayed with jittered points. The $y = 0$ marks the overall median and vertical bars show group-specific medians with 95% CIs. Group differences among HPAH, IPAH, and PVOD were tested with the Kruskal–Wallis test. A Cox proportional hazards

regression analysis was performed to estimate the hazard risk associated with plasma GDF15 levels in patients with PVOD, IPAH, and HPAH diagnosed at 16 years or older and for whom survival data were available. This was done in R version 4.3.2, using the survival (v3.8-3) and survminer (v0.5.0) packages with right censoring for transplant-free survival time from diagnosis. A P-value < 0.05 was considered statistically significant.

Author Contributions

A.P. and A.H. conceived and designed the study. A.P. E.DB., and P.G. performed experiments. E.DB. J.D., S.G., L.S.H., S.J.W., C.C., D.G.K., E.S., T.A., S.C., S.H., J.K, J.R.G., J.P.S., M.W, P.J.W., M.A.S., and M.T. enrolled patients, reviewed, and managed clinical data. A.P., E.DB. J.D., P.J.W., M.A.S., M.T. G.L, and A.H. analyzed data. A.P., E.DB. J.D., E.S., J.P.S., P.J.W., M.A.S., M.T. G.L., and A.H. drafted and revised the manuscript.

Acknowledgment

We thank Dr. Nicholas W. Morrell for his valuable discussions. We also acknowledge Bengisu Gur, Tarun Krishnan, Gopala Varadarajan, and Natalie Ojeda for their technical assistance. See Supplemental Acknowledgments for consortium details.

References

1. Shi G, Yue L, Tang Z, Wang Y, Hu X, and Tong Y. Serum growth differentiation factor 15 as a biomarker for malnutrition in patients with acute exacerbation of chronic obstructive pulmonary disease. *Front Nutr.* 2024;11:1404063.

2. Kim M, Cha SI, Choi KJ, Shin KM, Lim JK, Yoo SS, et al. Prognostic value of serum growth differentiation factor-15 in patients with chronic obstructive pulmonary disease exacerbation. *Tuberc Respir Dis (Seoul).* 2014;77(6):243-50.

3. Lv Z, Liang G, and Cheng M. Predictive Value of GDF-15 and sST2 for Pulmonary Hypertension in Acute Exacerbation of Chronic Obstructive Pulmonary Disease. *Int J Chron Obstruct Pulmon Dis.* 2023;18:2431-8.

4. Husebø GR, Grønseth R, Lerner L, Gyuris J, Hardie JA, Bakke PS, et al. Growth differentiation factor-15 is a predictor of important disease outcomes in patients with COPD. *Eur Respir J.* 2017;49(3).

5. Mutlu LC, Altintas N, Aydin M, Tulubas F, Oran M, Kucukyalin V, et al. Growth Differentiation Factor-15 Is a Novel Biomarker Predicting Acute Exacerbation of Chronic Obstructive Pulmonary Disease. *Inflammation.* 2015;38(5):1805-13.

6. Deng M, Bian Y, Zhang Q, Zhou X, and Hou G. Growth Differentiation Factor-15 as a Biomarker for Sarcopenia in Patients With Chronic Obstructive Pulmonary Disease. *Front Nutr.* 2022;9:897097.

7. Freeman CM, Martinez CH, Todt JC, Martinez FJ, Han MK, Thompson DL, et al. Acute exacerbations of chronic obstructive pulmonary disease are associated

with decreased CD4+ & CD8+ T cells and increased growth & differentiation factor-15 (GDF-15) in peripheral blood. *Respir Res.* 2015;16(1):94.

8. Agustí A, Celli BR, Criner GJ, Halpin D, Anzueto A, Barnes P, et al. Global Initiative for Chronic Obstructive Lung Disease 2023 Report: GOLD Executive Summary. *Eur Respir J.* 2023;61(4).

9. Galie N, Torbicki A, Barst R, Dartevelle P, Haworth S, Higenbottam T, et al. Guidelines on diagnosis and treatment of pulmonary arterial hypertension. The Task Force on Diagnosis and Treatment of Pulmonary Arterial Hypertension of the European Society of Cardiology. *Eur Heart J.* 2004;25(24):2243-78.

10. Rhodes CJ, Otero-Nunez P, Wharton J, Swietlik EM, Kariotis S, Harbaum L, et al. Whole-Blood RNA Profiles Associated with Pulmonary Arterial Hypertension and Clinical Outcome. *Am J Respir Crit Care Med.* 2020;202(4):586-94.

Supplementary Table 1 Demographic and clinical characteristics of study participants

Characteristics	Controls (n=68)	PVOD (n=26)	IPAH (n=98)	HPAH (n=42)	Other PH (n=52)	COPD (n=36)	*P*-value
Age, median years (IQR)	33.0 (28.0 – 49.0)	64.0 (45.75 – 70.0)	69.0 (44.75 – 73.0)	38.0 (30.0 – 54.75)	53.0 (46.0 – 68.75)	65.5 (62.25 – 68.75)	<0.0001
Sex: Male, n(%)	27(39.71%)	10(38.46%)	36(36.73%)	9 (21.43%)	21(40.38%)	21(58.33%)	
Sex: Female, n(%)	41(60.29%)	16(61.54%)	62(63.27%)	32(78.57%)	31(59.62%)	15(41.67%)	
Time from diagnosis to sampling, median years (IQR)	NA	1.83(0.40-2.09)	5.00(3.00-10.00)	5.93(2.99-9.81)	0.23(0.04-0.45)	NA	<0.0001
PVR, median dynes/sec/cm^{-5} (IQR)	NA	696.5 (528.0 – 1022)	731.3 (419.5 – 1108)	1292 (977.5 – 1688)	NA	NA	<0.0001
6MWD, median m (IQR)	NA	219.0 (78.0 – 281.0)	324.5 (215.0 – 429.8)	331.0 (281.5 – 390.0)	324.0 (278.0 – 371.0)	NA	0.0065
mPAP, median mmHg (IQR)	17.0 (12.5 – 21.0)	48.0 (39.5 – 54.5)	46.0 (39.0 – 61.0)	55.0 (51.75 – 65.25)	43.0 (26.0 – 53.0)	NA	<0.0001
NT-proBNP, median ng/L (IQR)	40 (39 – 41)	1688.0 (134.0 – 2801)	407.5 (167.8 – 1803)	2009 (1130 – 2962)	79.0 (27.0 – 253.5)	NA	<0.0001
Cardiac Index, median L/min/m^2 (IQR)	2.78 (2.39 – 3.14)	2.35 (1.765 – 2.865)	2.35 (1.775 – 3.037)	1.705 (1.466 – 2.071)	2.66 (2.0 – 3.48)	NA	<0.0001

Other PH includes patients with G1 PAH, excluding PVOD, IPAH, and HPAH, as well as G2-4 PH. IQR: interquartile range, Age: age at sampling, PVR: pulmonary vascular resistance, 6MWD: 6-minute walk distance, mPAP: mean pulmonary artery pressure, BNP: B-type natriuretic peptide, and CI: cardiac index. NA: data not available.

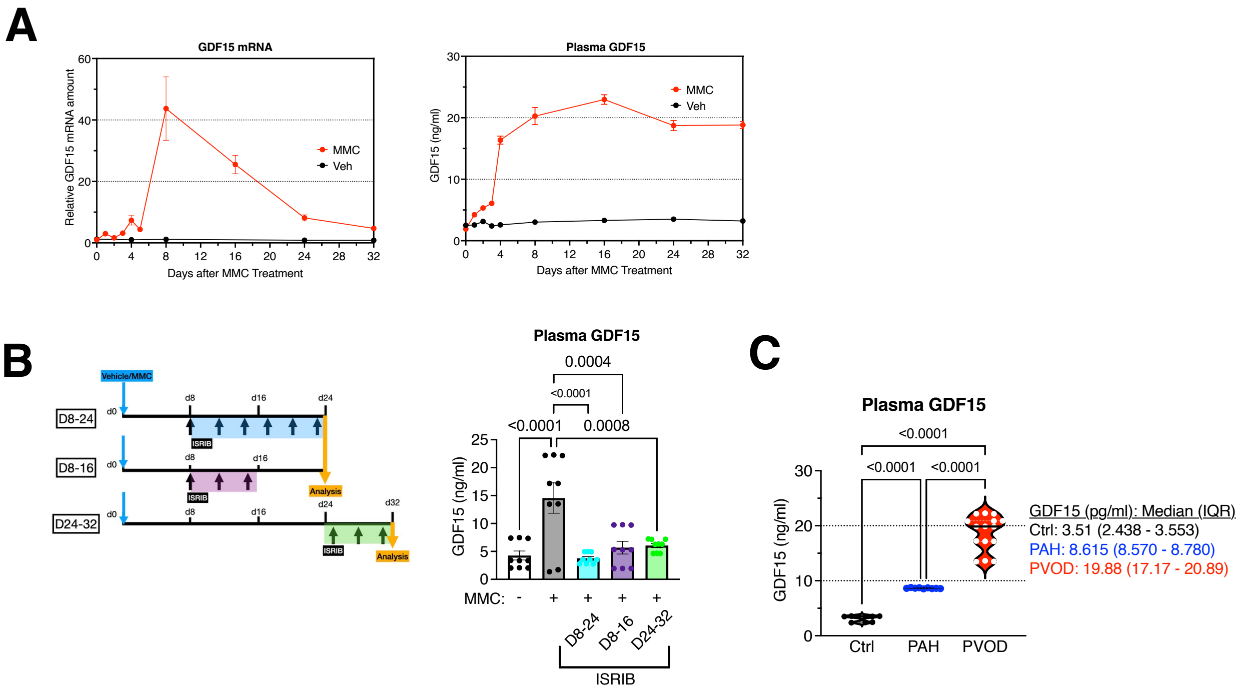

Supplementary Figure 1 Elevated plasma levels of GDF15 in an animal model of PVOD (A) GDF15 mRNA levels in the lungs (left) and plasma GDF15 concentrations (ng/mL) (right) were measured on day 0, 1, 2, 3, 4, 8, 16, and 32 by qRT-PCR and ELISA, respectively, after the treatment of rats with vehicle (black) or MMC (red) and shown as mean ± SEM. n = 6 per condition. **(B)** Schematic representation of three therapeutic ISRIB treatments in MMC-treated young adult rats (left). Plasma GDF15 concentrations (ng/mL) were measured 24 days after MMC administration and are presented as mean ± SEM (right). n = 9. **(C)** Violin plot of plasma GDF15 concentrations (ng/mL) in control (Ctrl), monocrotaline (MCT)-induced PAH, and MMC-induced PVOD model rats (left). Median (IQR) is shown (right). *n* = 9 (for Ctrl and PAH) and 15 (for PVOD). Data were analyzed by one-way ANOVA followed by Tukey's post-hoc test (**B** and **C**).

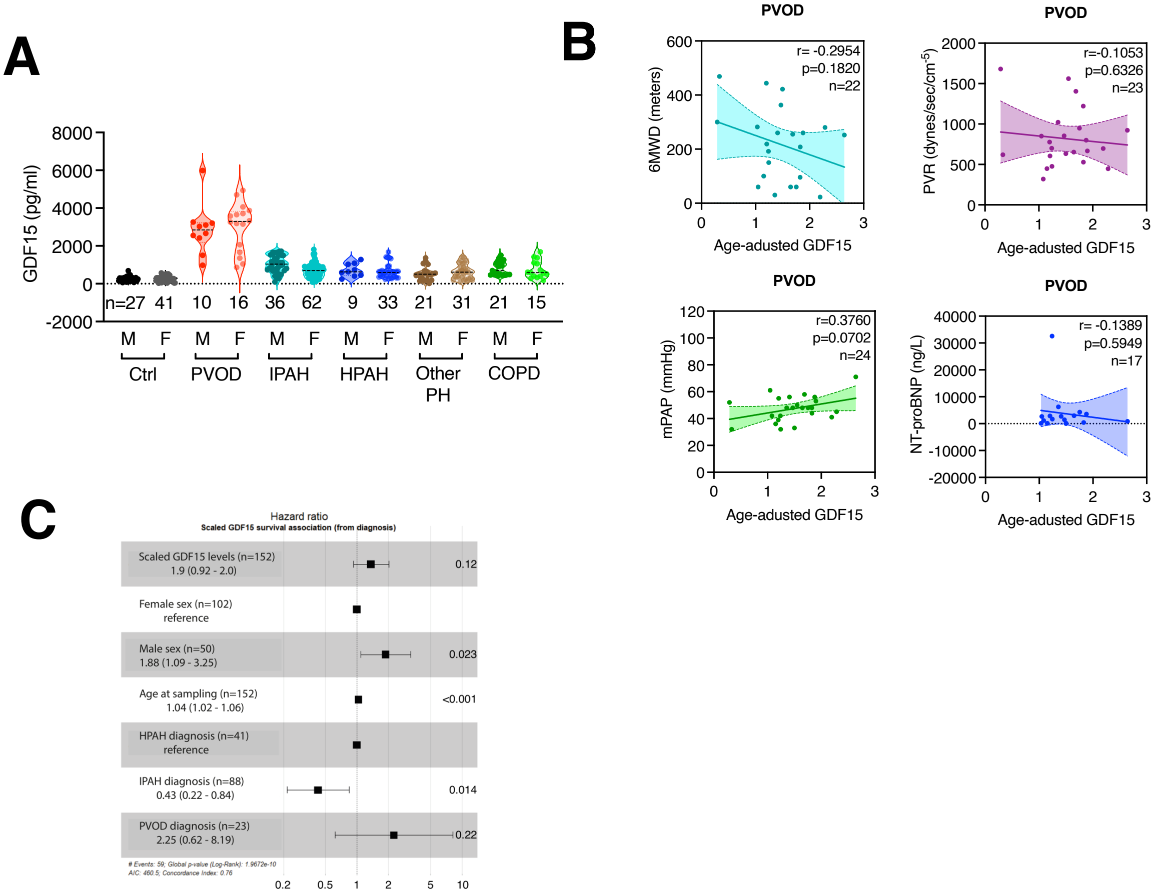

Supplementary Figure 2 Evaluating the potential of GDF15 as a diagnostic and prognostic biomarker for PVOD (A) The deviation of GDF15 levels from the median line was assessed in control individuals (Ctrl) and patients with PVOD, IPAH, HPAH, Other PH, and COPD, combining data from both the discovery and validation cohorts. M and F stand for male and female. The results are depicted using a two-tailed Mann–Whitney test. The solid circle represents the difference in median calculated by the Hodges-Lehmann method, and the lines show the 95% CI. **(B)** Pearson correlation coefficient (r), *P*-value, and sample size (n) are indicated for each analysis. Pearson correlation analyses were performed to assess the relationship between age-adjusted

GDF15 levels and various clinical parameters within the PVOD cohort. These parameters included: 6-minute walk distance (6MWD; light blue), PVR (purple), mPAP (green), and NT-proBNP (blue). The correlation coefficient (r), P-value, and sample size (n) are indicated for each analysis. **(C)** Cox proportional hazards model was used to assess transplant-free survival time in relation to age-adjusted GDF15 levels in the PVOD, HPAH, and IPAH cohorts after adjusting for age at sampling, sex, and disease subtype.

UK National Cohort Study of Idiopathic and Heritable PAH Consortium

Department of Medicine, University of Cambridge, Cambridge, UK: Nicholas W. Morrell, Mark R. Toshner, Stefan Gräf, Eckart MDD De Bie, Joseph Newman

Freeman Hospital, Newcastle, UK: Jim Lordan

Golden Jubilee National Hospital, Glasgow: Colin Church

Great Ormond Street Hospital, London, UK: Shahin Moledina, Lynsay MacDonald, Eleni Tamvaki, Anabelle Barnes, Victoria Cookson, Latifa Chentouf.

Royal Brompton Hospital, London, UK: Rosa DaCosta, Joy Pinguel, Natalie Dormand, Alice Parker, Della Stokes, Laura Price

Royal Free Hospital, London, UK: Dipa Ghedia, Yvonne Tan, Tanaka Ngcozana, Ivy Wanjiku, Gerry Coghlan

Royal Papworth Hospital, UK: Joanna Pepke Zaba, Katherine Bunclark, Gary Polwarth

Royal United Bath Hospitals, Bath, UK: Rob V. Mackenzie Ross, Jay Suntharalingam, Mark Grover, Ali Kirby, Ali Grove, Katie White, Annette Seatter.

Sheffield NIHR Clinical Research Facility, Royal Hallamshire Hospital, Sheffield, UK: Amanda Creaser-Myers, Sara Walker, Stephen Roney, Roger Thompson, Alexander Rothman

National Heart and Lung Institute, Imperial College London, London, UK: Charles A. Elliot, Athanasios Charalampopoulos, Ian Sabroe, Abdul Hameed, Iain Armstrong, Neil Hamilton, Stephen J Wort, Luke S Howard, Martin Wilkins, Christopher Rhodes, Allan Lawrie

University of Sheffield, Sheffield, UK: Andrew J. Swift, James M. Wild, Dennis Wang, David G. Kiely, Robin Condliffe
